# Risks for STIs/HIV infection among Madawalabu University students, Southeast Ethiopia: a cross sectional study

**DOI:** 10.1186/1742-4755-10-38

**Published:** 2013-08-01

**Authors:** Tesfaye Setegn Mengistu, Abulie Takele Melku, Nagasa Dida Bedada, Begna Tulu Eticha

**Affiliations:** 1 Department of Nursing, College of Medicine and Health Sciences, Madawalabu University, Bale Goba, Ethiopia; 2 Department of Public Health officer, College of Medicine and health Sciences, Madawalabu University, Bale Goba, Ethiopia; 3 Department of Medicine, Microbiology, Parasitology and infectious diseases unit, College of Medicine and Health Sciences, Madawalabu University, Bale Goba, Ethiopia

## Abstract

**Introduction:**

In developing nations, the spread of STIs/HIV infection continues to affect millions of young and productive population. In Ethiopia youths including university/college students are at greater risk of STIs including HIV infection often due to many risky sexual behaviors. Although there are some anecdotal evidences suggesting widespread unsafe sexual practices among university students, the paucity of research finding, especially in newly established public universities are the major bottle necks to commence feasible interventions. Therefore, this study was designed to assess the magnitudes and factors associated with risks for STIs/HIV infections among Madawalabu university students in Southeast Ethiopia.

**Methodology:**

An institution based cross sectional study was conducted from May-June 2012. A total of 390 students were selected using stratified then simple random sampling method. Descriptive statistics, binary logistic and multivariable logistic regression analyses were employed to identify factors associated with risks for STIs/HIV infection.

**Result:**

Combined risk measure showed that 51.4% of students were at risk of having STIs and/or HIV infection. Practicing casual sex/sex for benefits with first sexual partner (OR = 3.9[95%C.I: 1.86-8.03]), life time multiple sexual partner (had more than three sexual partners) (OR = 2.7[95%C.I: 1.13-6.28]), and number of sexual partners in the last 12 months (four and above) (OR = 4.8[95%C.I: 1.77-13.53]) showed statistically significant association with risks for STIs and/or HIV infection. Practicing casual sex/ sex for any benefit with their first sexual partner (AOR = 3.9 [95%CI: 1.80-8.50]) and multiple sexual partners in the last 12 months (four and above) (AOR = 3.7 [95%C.I: 1.15-11.80]) were found to be the independent predictors of risks for STIs and/or HIV infection.

**Conclusion:**

This study has identified risks and risk sexual behaviors for STIs and/or HIV infection on university students. The knowledge of the students towards STIs and/or HIV is unsatisfactory. More than half of the students were at risk for STIs and/or HIV infection. Casual/benefit based sexual relationship with first sexual partner and having multiple sexual partners (≥4 sexual partners) in the last 12 months were independent predictors of STIs and/or HIV infections. Therefore, university based, risk reduction and behavior change focused interventions are recommended.

## Introduction

The HIV pandemic remains the most serious infectious disease and a challenge to public health intervention. Globally, the epidemic has regional, population and gender disparity. Sub-Saharan Africa remains the most affected region in the global AIDS epidemic in which Ethiopia is one of the hardest hit sub-Saharan countries. Most of the deaths attributed to HIV/AIDS were because of inadequate access to HIV prevention and treatment services. It disproportionately affects youth generation which is almost half of the global population and leaves countries, communities, and households without productive people. Therefore its impact is not limited only to health problem [1-7].

Not only from HIV infection, adolescents are being affected and infected from STIs or both. The prevalence of STIs is highest in developing nations. Because of young people’s risky behaviors and low use of preventive behaviors and/or services, the epidemic has fastest and growing effect on youth population. It is estimated that in developing countries, one in 20 youth contracts an STI each year. Young people especially who are unlikely to have access to quality health care services such as university students have higher rates of STIs but they have largely been ignored [4,8]. University/College students are often viewed as being at higher risks to acquire STIs/HIV infection and they are categorized under the Most At Risk Population Segments (MARPS) due to their inclination to be engaged in risky sexual behavior and their sense of non-vulnerability [9].

In universities- in countries with high HIV/AIDS prevalence-significant proportion of their students and staffs might have been infected with HIV [10]. In Ethiopia, sexual activity with all the associated risks such as STIs including HIV infection will sets on during adolescence period. Ethiopia is a country with highest HIV infection rate in the world. It is estimated that 20% of youth/adolescent population is found in the age group of 15–24 years of which 2.9% is HIV infected. Youths and adolescents are at greatest risk of STIs/HIV infection because of individual, biological and cultural factors. Girls start having sex earlier than boys; those female youths/adolescents who would have sexual relation with older men usually don’t have experience in negotiating safer sex [7,10-12].

A study conducted in China showed that 17.6% of males and 8.6% of females were sexually active. The mean age at first sexual debut was 19.4 and 19.7 for males and females, respectively. But the proportion of male students who had experienced sex before joining university was significantly higher than female students [13]. Multiple sexual partner rate in Nigerian university students was 3.5 sexual partners on average, while 63% of Togolese university students had more than one sexual partner. Similarly, in Malawi University, 40.4% of students had reported multiple sex partners in the last 12 months [14-16].

In Ethiopia, significant proportion of the population, particularly the youths have been identified at risk of HIV infection despite high level of knowledge about HIV/AIDS [17]. In Jimma University, 97% of students do have knowledge on HIV/AIDS, voluntary counseling and testing. But, 56.3% of students involved in unprotected sex with casual partners and failed to recognize that they are at risk of HIV infection. Twelve percent (12.2%) of students were sero positive. Similarly, in Gonder University, 23% of university students had reported sexual contact with prostitutes and only 37.1% of them used condom. In a study conducted in Wolaita Sodo University, 97.3% of students had good knowledge on HIV/AIDS [18-21].

A study conducted in Bahir Dar University, has reported that 69.1% of students were sexually active; of which 25.3% started sexual intercourse before the age of 18 years. The study also indicated that 27.8% of students have had multiple sexual partners and 34.4% had practiced unprotected sex. About eight percent (7.8%) of students had sex with commercial sex workers [22].

In Haromaya University, 41.2% of students were sexually active, and 27.8% of students had multiple sexual partners (2–5 partners on average). In this study, (39.9%) of the students had reported sexual contact with commercial sex workers (CSW) while 23.51% of the students reported to have sex with casual friend [23].

Recognizing the socio-demographic and health impact of STIs and/or HIV infection on major and productive population and underscoring that universities are important fronts in the fight against STIs and/or HIV transmission, different levels of interventions have been implemented to reach university students [24,25]. But in recently established public universities, there has been paucity of researches to undertake informed institution based risk reduction interventions. Therefore, this study is aimed to assess risks for STIs/HIV infection and associated factors among Madawalabu University students.

## Methods

### Study setting and sample

An institution based cross sectional study was conducted in Madawalabu University from May to June/2012. Madawalabu University was established in 2007 and located in Bale Zone, Robe Town 430 KM from Addis Ababa, Southeast of Ethiopia. It is one of the newly established public higher educational institutions. The university has two campuses (the main campus in Robe town and College of Medicine and Health Science in Goba town). It has nine schools, one college and one institute. Currently, a total of 10,317 students- of which 5,275 are regular students- are enrolled.

The sample size was determined using single population proportion formula assuming 50% expected prevalence of risks for STIs/HIV infection and by adding 10% of the calculated sample size for non-response. A total of 422 students were selected using stratified sampling technique. All academic schools and colleges were stratified as “None health and health” and six [6] none health schools and a health college, totally seven schools were selected using simple random sampling technique. The total number of study participants for each selected college or schools was obtained from the registrar and alumni directorate office and the total sample size 422 was allocated proportionally to size of each of the selected six schools or college and then to each department. Finally, simple random sampling was employed to select students from each department. The structured Amharic version questionnaire was distributed to students in the class rooms. Trained academic staffs were recruited to facilitate the data collection process.

### Measurements

Data were collected using a questionnaire adapted from EDHS, EPHA, WHO [4,7,26,27]. The adapted questionnaire was contextualized to the local situation and to the research objectives. Structured self-administered questionnaire was prepared in English, translated to Amharic and the later version was used to collect the data. To measure risks for STIs/HIV infection, students were asked lists of “Yes” “No” and multiple answer questions. Undesired sexual and behavioral responses were combined to measure the risks for STIs/HIV infection and indexed combined risk measure was used to dichotomize the risk level of students.

### Data analysis

The data were checked for completeness, inconsistencies, cleaned then coded and entered in to SPSS for windows version 16.0. Data exploration was made to see wild values which may affect the overall result and interpretation. Descriptive statistics was computed to determine the magnitude of STIs/HIV infection risks. Binary logistic regression analysis was carried out to determine the differential of risks for STI/HIV infection with independent variables separately.

Then, to control the confounding effect of other variables and to determine independent predictors of risks for STI/HIV infection, multivariable logistic regression analysis was carried out by taking significant variables in the binary logistic regression model. Statistical significance was declared at P < 0.05. The strength of statistical association was measured by adjusted odds ratios and 95% confidence intervals.

Letter of ethical approval was received from Madawalabu University Research and Community Service Directorate Office (RCSDO). Informed verbal consent was secured from study participants. The purpose of the study, potential risk and benefits of participating in the study and the right of the participants to withdraw from the study any time was explained. The participants were also assured about the confidentiality of the data.

## Result

From the total of 422 identified regular students, 390 were responded the questionnaire and included in the analysis. This made the response rate 92.4%. The mean (±SD) age of students was 21.3 (±1.5) years. Majority (90.3%) of students were in the age range of 20–24 years. Seventy nine percent of students were male while 51.2% of the respondents were orthodox by religion. Oromo constitute the largest ethnic group (63.7%) followed by Amhara (20.5%). Majority (95.6%) of students were living in campus (Table 1).

**Table 1:** Socio-demographic characteristics of undergraduate students in Madawalabu University, 2012

**Socio-demographic variables**	**Number**	**Percent**
Age of participants (years)		
≤19	23	5.9
20-24	352	90.3
25-29	15	3.8
Mean(SD)	21.3 (±1.5) years	
Sex of participants		
Male	309	80.3
Female	76	19.7
Marital status		
Never married	350	91.1
Currently married	10	2.6
Have constant sexual partner	24	6.3
Religion		
Orthodox	185	51.2
Protestant	87	24.1
Muslim	80	22.2
Others®	9	2.5
Ethnicity		
Oromo	230	63.7
Amhara	74	20.5
Tigrie	31	8.6
Other*	26	7.2
Place of residence		
In campus	368	95.6
Out of campus	17	4.4
Monthly allowance from family		
Yes	325	83.8
No	63	16.2
Perceived family economic status		
Rich	9	2.4
Medium	267	70.3
Poor	77	20.3
Do not Know	27	7.0

® Adventist, wakefeta * Siltie, Berta, some other from Southern nations and nationalities.

### Risks for STIs/HIV infection

**Figure 1:**
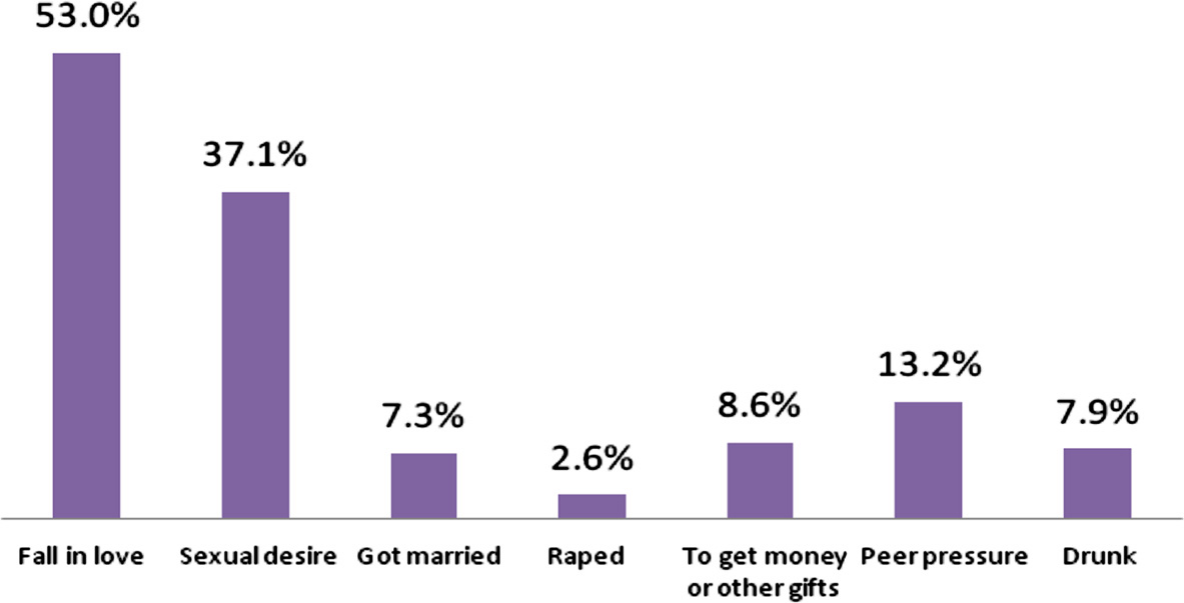
Reasons given by sexually active university students to have sex for the first time, Madawalabu University, 2012.

Of the total study participants, 160 (42.3%) of the students were sexually active (male; 90.5% Vs females; 9.5%). The mean as (±SD) reported age at first sex was 18.6 (±2.2) years. Of the total sexually active students (34.7%) had initiated sex after joining university. Fifty six percent (56.2%) of students practiced unprotected sex. Of the total study population, 51.4% have risks for STIs/HIV infection (Table 2). Majority (53.0%) of sexually active students gave falling in love as a reason to initiate sex. Thirteen percent had reported that they initiated sex due to peer pressure while 8.6% of students practiced sex to get money or other benefits from their sexual partners (Figure 1).

**Table 2:** Distribution of risk sexual behaviors, knowledge and measured risk for STIs/HIV infections among sexually active Madawalabu University students (n = 160), 2012

**Variables**	**Frequency**	**Percentage**
Place for first sexual activity		
Before joining campus	94	65.3
After joining campus	50	34.7
Relation with first sexual partner		
Casual	40	27.6
Permanent sexual partner	84	57.9
Benefit based relationship	14	9.7
Spouse	7	4.8
Age difference with first sex partner		
More than 10 years older	15	10.4
5-10 years older	19	13.2
1-5 years older	26	18.1
< 5 years younger	24	16.7
We were in similar age	60	41.7
Number of life time sexual partners		
One	84	52.5
Two	18	11.2
Three	21	13.1
More than three	37	23.1
Condom use for firs sexual activity		
Yes	63	43.8
No	81	56.2
Number of sexual partners in the last 12 months		
One	94	69.6
Two	20	14.8
Three	12	8.9
Four and above	9	6.7
Condom use for last sexual activity		
Yes	75	56.0
No	59	44.0
History of sex with CSW* (For male students)		
Yes	30	24.0
No	95	76.0
Have STIs syndrome		
Yes	29	9.4
No	279	90.6
Measured overall risks for STIs/HIV infection		
Yes	72	51.4
No	68	48.6
Knowledge level of respondent (mean score = 7.56)**		
Poor knowledge (below mean score)	92	57.5
Knowledgeable (above mean score)	68	42.5

*commercial sex worker, **cronbach’s alpha 61.6% (internal consistency measure of knowledge items).

#### Factors associated with risks for STIs/HIV infection

To identify factors associated with risks for STIs/HIV infection, the Hosmer-Lemeshow statistic at *P > 0.05* and model chi-squares were checked to assess the data fit for logistic regression. On binary logistic regression analysis, casual/benefit based relationship, having multiple sexual partners (i.e. three or more) and number of sexual friends in the last 12 months (four and above) were associated with risks for STIs and/or HIV infection.

Type of sexual relation with first sexual partner (casual/benefit based sexual relationship) has statistically significant association with the risks of STI and/or HIV infection (OR = 3.9[95%C.I: 1.86-8.03]). Similarly, the number of life time sexual partner (three or more sexual partners) was found to be statistically significant with the outcome variable (OR = 2.7[95%C.I: 1.13-6.28]). Number of sexual partners in the last 12 months (four and above) was statistically associated with risks for STIs/HIV infection (OR = 4.8[95%C.I: 1.77-13.53]) (Table 3).

**Table 3:** Factors associated with risks for STIs/HIV infections among Madawalabu University sexually active students (n = 160), 2012

**Variables**	**Risk for STIs/HIV**		**COR [95%C.I]**
	**Yes (%)**	**No (%)**	
Place for first sexual activity			
Before joining campus	42(51.9)	39(48.1)	1.1[0.51-2.20]
After joining campus	25(51.0)	24(49.0)	1.0
Relation with first sexual partner			
Casual /benefit based relationship	19(32.8)	39(67.2)	3.9[1.86-8.03]^*#*^
Permanent friend/spouse	47(65.3)	25(34.7)	1.0
Age difference with first sex partner			
> 10 years older	5(33.3)	10(66.7)	2.2[0.67-7.27]
5-10 years older	12(60.0)	8(40.0)	0.7[0.26-2.07]
1-5 years older	9(45.0)	11(55.0)	1.4[0.50-3.75]
< 5 years younger	10(58.8)	7(41.2)	0.8[0.26-2.31]
We were in similar age	31(52.5)	28(47.5)	1.0
Number of life time sexual partners			
One	41(60.3)	27(39.7)	1.0
Two	10(58.8)	7(41.2)	1.1[0.36-3.13]
Three	9(40.9)	13(59.1)	2.2[0.82-5.84]
More than three	12(36.4)	21(63.6)	2.7[1.13-6.28]^#^
Condom use for firs sexual activity			
Yes	27(52.9)	24(47.1)	1.0
No	39(47.6)	43(52.4)	1.3[0.62-2.50]
Number of sexual partners in the last 12 months			
One	51(60.7)	33(39.3)	1.0
Two	9(47.4)	10(52.6)	1.7[0.63-4.67]
Three	6(50.0)	6(50.0)	1.5[0.46-5.20]
Four and above	6(24.0)	19(76.0)	4.8[1.77-13.53]^*#*^
Condom use (last 12 month)			
Yes	37(56.1)	29(43.9)	1.0
No	27(45.8)	32(54.2)	1.5[0.75-3.06]
History of sex with CSW* (For male students)			
Yes	13(43.3)	17(56.7)	1.6[0.69-3.62]
No	52(54.7)	43(45.3)	1.0

^***#***^Significant at p < 0.05 (2-tailed), *commercial sex workers.

## Discussion

This study has revealed risky sexual behaviors for STIs and/or HIV infection with their magnitudes such as casual first time sex (27.6%); practicing sex for any kind of benefit (9.7%), wide range of age difference (10 years or more) with first sexual partner (10.4%), life time multiple sexual partners (47.4%) (Table 2). In this study, the multivariable logistic regression analysis (adjusted for potential confounders) showed that casual/benefit based sexual activity with first time sexual partner (AOR = *3.9[95%CI: 1.80-8.50]*) and having four and above sexual partners in the last 12 months (AOR = 3.7[95%*C.I*: *1.15-11.80*]) were independent risk predictor of STIs/HIV infection (Table 4).

**Table 4:** Multivariable logistic regression model showing predictors of risk for STIs/HIV infections among sexually active Madawalabu University students (n = 160), 2012

**Variables**	**Risk for STIs/HIV**		**AOR [95%C.I]**
	***Yes (%)***	***No (%)***	
Relation with first sexual partner			
Casual/benefit based relationship	19(32.8)	39(67.2)	***3.9[1.80-8.50]®***
A permanent friend/Spouse	47(65.3)	25(34.7)	1.0
Number of life time sexual partners			
One	41(60.3)	27(39.7)	1.0
Two	10(58.8)	7(41.2)	1.0[0.30-3.30]
Three	9(40.9)	13(59.1)	1.6[0.50-5.32]
More than three	12(36.4)	21(63.6)	1.4[0.46-4.00]
Number of sexual partners in the last 12 months			
One	51(60.7)	33(39.3)	1.0
Two	9(47.4)	10(52.6)	1.4[0.45-4.54
Three	6(50.0)	6(50.0)	1.4[0.26-7.75]
Four and above	6(24.0)	19(76.0)	***3.7[1.15-11.80]®***

**®**Significant at p < 0.05(2-tailed) *AOR* Adjusted Odds Ratio.

From the total students who reported life time multiple sexual partners, those who reported two sexual partners were (11.2%), three sexual friends (13.1%) and 23.1% of students had more than three sexual partners in their life time. The proportion of life time multiple sexual partner is almost similar with the finding of the study conducted in Haromaya University (35.4%). But the figure is by far greater than the findings in Jimma University (28.9%) and Bahir Dar University (27.8%). The figurative difference could be due to the difference in sample size and comprehensive university based behavioral change interventions in the above mentioned universities. But a study in Wolaita Sodo University has reported life time multiple sexual partner rate of 70.6%. The study conducted in Nigerian University, students have reported an average of 3.5 sexual partners. A study conducted in Benin, Togolese university, showed that 63% of students had more than one sexual partner [14,15,20-23].

In this study, 30.4% of students have had multiple sexual partners in the last 12 months. A similar study in Malawi University showed that 40.4% of the students reported multiple sex partners in the last 12 months. In our study, 44.0% of the students practiced unprotected sexual intercourse in the last 12 months. This rate of unprotected sexual practice is nearly similar with the finding in Malawi University (37.4%) and Bahir Dar University (34.4%). But the rate of unprotected sex with casual partners in Jimma University looks somehow greater (56.3%) [16,18,19,22]. This difference might be attributed to socio-demographic and sample size differences between the participants and studies respectively.

In this study, 24.0% of sexually active male students reported ever had history of sexual contact with commercial sex workers (CSW). Similarly, in Gonder University, 23% of university students had reported sexual contact with prostitutes while a study in Haromaya University reported that 39.9% of sexually active male student had sexual contact with commercial sex workers. But in Bahir Dar University, 7.8% of students reported sexual contact with commercial sex workers [20,22,23]. Concerning the factors associated with the risks for STIs/HIV infection, 34.7% of the sexually active students had practiced their first sexual intercourse after they joined university. This could be due to low knowledge of student (39.2%) regarding risks for STIs/HIV infection.

The current study has indicated that having sex with casual sexual friend/ sex for any benefit has a statistically significant association with risks of STIs and/or HIV infection. In line with its theoretical/clinical significance, those students who have had casual/benefit based sexual intercourse with their first sexual partner were about 4 times more likely to have risks for STIs and/or HIV infection when compared to those who have permanent sexual friend/spouse (OR = 3.9[95%C.I: 1.86-8.03]). Similarly, the number of life time multiple sexual partners has statistical association with the risks for STIs and/or HIV infections. Therefore, students who have had more than three sexual partners in their life time were about 3 times more likely to have risks for STIs/HIV infection when compared to their counter parts who have had one sexual partner (OR = 2.7[95%C.I: 1.13-6.28]).

The number of sexual partners in the last 12 months (≥ 4) showed statistical associated with risks for STIs/HIV infection with an increased odds ratio as compared to life time multiple sexual partners (OR = 4.8[95%C.I: 1.77-13.53]). Students those who have had four or more sexual partners in the last 12 months were about 5 times more likely to have STI/HIV infection risks when compared with their counter parts who have one sexual partner.

The multivariable logistic regression analyses showed that practicing casual/benefit based sexual activity with first time sexual partner and having four and above sexual partners in the last 12 months were statistically significant predictors of STIs/HIV infection risks.

Given that, life time multiple sexual partner is in the model, those who practiced casual sex/ sex for any benefit with their first sexual partner were 4 times more likely to have risks for STIs/HIV infection when compared to those students who practiced their first sex with their permanent sexual friends/spouse (AOR = *3.9(95%CI: 1.80-8.50*). Similarly, those students who had multiple sexual partners in the last 12 months (four and above sexual partners) were *3.7* times more likely to have risks for STIs/HIV infections when compared to their counter parts who have had only one sexual partner in the last 12 months (AOR = 3.7[95%C.I: *1.15-11.80*]).

The use of validated questionnaire and index combined risk measure could be the strengths of this study. However, students might report most desired behaviors and recent event which might introduce social desirability and recall biases respectively. We could not measure reason behind risky sexual behaviors qualitatively. In this study the cronbach’s alpha for knowledge questions as below the acceptable range which would affect generalization of the study with regards to knowledge of students only. Finally, this study is also limited to those missing data, thus interpretation of the finding shall take the missing date in to account.

## Conclusion

The study identified different risk sexual behaviors that predispose university students for STIs and/ or HIV infection with their respective magnitudes. The knowledge of the students towards STIs and/HIV is unsatisfactory. Most of the risks for STIs and/HIV infection were identified with an attention seeking magnitudes. More than half of students were at risk for STIs and/ or HIV infection due to their varying levels of sexual behaviors such as practicing multiple sexual partners(both life time and in the last 12 months) , casual sexual intercourse/sex to get any benefit, sex with risk groups(CSW). Unprotected first time sexual act was reported-which might be practiced after students has joined university- which could be evidenced by one third of the students had practiced their first sexual intercourse after joining university.

Generally this study has identified that more than half of the students were at risk for STIs and/or HIV infection and casual/benefit based sexual relationship with first sexual partner and having multiple sexual partners in the last 12 months (four and above sexual partners) were independent risk predictors of STIs and/or HIV infections. Therefore, strengthening student clubs, designing and implementing awareness rising and risk reduction activities and promoting peer education are recommended interventions.

## Competing interests

The authors declare that they have no competing interests.

## Authors’ contributions

TS conceived and designed the study, performed analysis and interpretation of data and drafted the manuscript. AT assisted with the design conception, analysis, and interpretation of data. ND assisted the study design, data interpretation and critically reviewed the manuscript. BT assisted data collection, entry and reviewed the manuscript. All authors read and approved the final manuscript.
